# Case of Carcinoma of Intestine

**Published:** 1878-04

**Authors:** 


### CASE OF CARCINOMA OF INTESTINE.

By Dr. Dewey.

A. C., female, 50 years old, married, and of Swedish birth, was admitted to the Illinois Northern Hospital for the Insane April 19, 1875, suffering from secondary dementia, with predominant religious delusions. Although abominably filthy in her habits, she considered her person holy and sacred. Her previous history could not be ascertained.

No marked change in mental or physical condition was observed until February, 1877, when she began to lose flesh, strength and appetite. She seemed to suffer griping pains in the bowels, and usually sat upon the floor, her head bowed, her knees drawn up, her hands clasped tightly across her stomach; yet she never acknowledged any pain.

Feb. 26.—Jaundice was observed. Patient refused to answer questions and violently resisted all attempts at physical examination.

March 16.—Vomiting and constipation had become constant symptoms. The abdomen was everywhere hard and tense; evacuations induced by enemata were of grayish color and very offensive. There were frequent periods of extreme prostration.

March 21.—Patient's actions indicate severe pain in the right hypochondriac region; pulse is rapid and thready.

March 22.—There is pain in the left side, also; her general condition is unimproved; she vomits very offensive greenish fluid.

March 24.—We find a soft, fluctuating tumor 1½ inches in diameter, situated in the median line three inches above the umbilicus. There is general flatness on percussion over the epigastrium, but no distension. Death occurred March 25.

An examination 18 hours later showed the abdominal walls adherent to the transverse colon and to one or two folds of the small intestine on the left of the median line, over an irregular space four or five inches in diameter. The tumor above mentioned protruded between the recti muscles, covered only by the integument and peritoneum. It was of irregularly oval shape, four by six inches in extent, involving the walls of the stomach, transverse colon, small intestines and abdominal coverings.

The stomach was eroded upon its lower internal surface, its walls thickened and adherent to the transverse colon. An irregular, sinuous canal led from one to the other, about two and one-half or three inches in length. The surfaces of the different viscera presented many slight patches of plastic exudation, while numerous flocculi of gray lymph floated in the serum which partly filled the abdominal cavity. The intestines contained much ichorous pus. No enlarged glands nor other deposits were found. A portion of the tumor was sent to Dr. Danforth for microscopic examination.

*Microscopy.*—The specimen presented, under the microscope, an alveolated network of connective tissue, containing in the interspaces dense groups of atypical cells. Their sections showed the usual cylindriform cell-growth of cancer. The case was one of cancerous infection of the lymphatic vessels and glands.

